# Agile analytics to support rapid knowledge pipelines

**DOI:** 10.1038/s41746-020-00309-z

**Published:** 2020-08-19

**Authors:** Wade L. Schulz, Joseph C. Kvedar, Harlan M. Krumholz

**Affiliations:** 1 Department of Laboratory Medicine, Yale School of Medicine, New Haven, CT 06510 USA; 2 Center for Outcomes Research and Evaluation, Yale New Haven Hospital, 1 Church Street, New Haven, CT 06510 USA; 3 Partners HealthCare and Harvard Medical School, Boston, MA 02115 USA; 4 Section of Cardiovascular Medicine, Department of Internal Medicine, Yale School of Medicine, New Haven, CT 06510 USA; 5 Department of Health Policy and Management, Yale School of Public Health, New Haven, CT 06510 USA

**Keywords:** Health services

Windows of opportunity can open during a global health threat and transform how we solve problems and generate knowledge in medicine. In an industry that can take years to change, the COVID-19 pandemic has led to massive international data collection and research efforts that would have seemed impossible before. The resistance to adopting new technology, data sources, and analytical methods has begun to yield to the urgency of the moment. Access to information has transformed the literature, with numerous publications that leverage digital health data, such as that from the electronic health record, to generate the evidence needed to better understand SARS-CoV-2 infection, treatment, and outcomes^1,2^.

The paper by the International Consortium for Clinical Characterization of patients with SARS-CoV-2 by EHR (4CE) describes just such an extraordinary response to the COVID-19 pandemic^3^. An international collaboration of 96 hospitals across five countries was assembled to create the means to produce insights through a decentralized platform. The group leveraged real-world data to create a diverse, international cohort of patients to assess clinical characteristics in patients with SARS-CoV-2 infection. Their approach has positioned them for rapid data collection and evidence generation, and the authors have used the data in a way that is fit-for-purpose for the study being conducted.

The appropriate scoping of methods for real-world data studies is critical for the use of these valuable, emerging data sets. We have just witnessed the retraction of two data-driven COVID-19 studies from two of the most prominent clinical journals because of uncertainty about the provenance and quality of the data^4,5^. But the issue goes far beyond these two papers, as data provenance and analytical approaches are often not appropriately detailed in the methods of scientific publications. The analogy to laboratory science would be investigators using complex biological techniques with no documentation of approach beyond the name of the method being used. For example, imagine an investigator publishing the findings of a study based on genetic sequencing, and the reported approach was that “DNA was sequenced” with no discussion of the technology, methods, or reagents used.

Observational studies and real-world data have an important role in biomedical research. They can enable more rapid, substantial, and generalizable results than randomized controlled trials. But they also come with limitations: they are more prone to bias and cannot be used to determine causality. Each type of study is an important part of the evidence base to guide decision making, and neither should be seen as a singular gold standard. While the pandemic places extra urgency on answering seemingly basic questions, a rigorous scientific approach is needed regardless of the type of study. With data-driven studies, especially those based on observational and real-world data, comes a responsibility to vouch for every step of the data pipeline, to understand the strengths and limitations of the data, and to appropriately interpret the findings in the context of the information available.

What is needed are investigators who are trained in the design of studies based on real-world and observational data, with routinely used best practices to analyze the data and report not just results, but also the methods used to generate them. Several groups are pushing for better methods in this area, from standards for data modeling to observational data analysis. Over the last decade, groups of investigators have coalesced around the use of common data models, such as those described in the 4CE article, which rely on Integrating Biology and the Bedside (i2b2)^6^, and international communities such as Observational Health Data Sciences and Informatics^7^. The latter aims to develop a data model and associated tools and best practices for observational research. These approaches for data standardization are now being leveraged for national consortia related to COVID-19 research, such as the National Institutes of Health’s National Center for Advancing Translational Sciences, which has launched a collaborative among academic centers called the National COVID Cohort Collaborative (N3C)^8^. However, a common data model, while essential, only solves a sliver of the potential issues for data-driven research.

The 4CE Consortium has set an example for the rapid deployment of a multinational group and addresses many potential concerns related to the design and reporting of such a study. First, the ethics statement defining the approach to institutional review board approval is noted in the methods and, as only aggregate data were centralized, patient privacy was further protected. The study clearly defines the phenotype, patients with a positive PCR test for SARS-CoV-2 and highlights the limitations, in that the cohort included outpatient, emergency department, and inpatient populations that could not be separated at the time of analysis. Furthermore, the authors detail the methods used to generate aggregate data and provide insights into the data quality by clearly describing the drop-off in test result frequency over time. Finally, the conclusions they have drawn are appropriate for the data set and highlight the limitations of such a study. They are now poised for an array of important future contributions.

The COVID-19 pandemic has created an opportunity and a hazard. The opportunity, as seized by the 4CE Collaborative, is to actualize an approach to agile analytics to support rapid knowledge generation. The ability to implement such a pipeline relies on ideas that have been years in the making and leverage infrastructure already in place. Data have the potential to transform the research enterprise, but not without access to data. However, the hazard is that some people who gain access to extensive data lack the skills to evaluate, analyze, or interpret it, and journals, in their haste, are often not prepared to adequately review such studies.

In our eagerness to use real-world data, we must insist on ensuring that the scientific process is followed, that high-quality data are used, and that methods are transparent and reproducible. The bar for this work needs to be set high, but we must also be able to move quickly. Examples such as the 4CE Collaborative show that both can be achieved. Now is the time to keep the momentum going.
